# Circadian and behavioural responses to shift work-like schedules of light/dark in the mouse

**DOI:** 10.1186/2049-9256-1-7

**Published:** 2013-05-29

**Authors:** Niall M McGowan, Andrew N Coogan

**Affiliations:** Department of Psychology, National University of Ireland Maynooth, County Kildare, Ireland

**Keywords:** Circadian, Shift work, Mouse, Entrainment

## Abstract

**Background:**

Disruption of circadian rhythms is associated with several deleterious health consequences and cognitive impairment. It is estimated that as many as one in five workers are exposed to this risk factor due to experiencing some degree of chronodisruption by way of recurring patterns of shift work. It is not presently clear therefore how efficiently the mammalian circadian system entrains to alternative light/dark cycles such as those found in shift work schedules.

**Methods:**

The present study examines male CD-1 mice exposed to three different paradigms of rapidly rotating shift work-like light/dark manipulations compared to control animals maintained on a standard 12:12 h light/dark cycle.

**Results:**

Analysis of circadian patterns of behaviour under such conditions reveals that for fast rotating schedules of light/dark there is minimal circadian entrainment. Further, when placed in constant conditions after a period under the “shift work” lighting conditions there were changes to circadian period associated with the shift work schedules. In contrast to previous studies the shift work-like conditions did not produce changes in animal body-weight. Behavioural testing suggests possible anxiogenic and hyperactive outcomes dependent on rotation speed as animals displayed open field thigmotaxis and hyperlocomotion.

**Conclusion:**

These results indicate that exposure to alternating patterns of light and dark as experienced by millions of shift workers may produce long-lasting changes in both mammalian circadian and neurobehavioural systems.

## Background

Circadian rhythms are endogenous rhythmic processes which display periods of approximately 24 hours in the absence of environmental time cues. In nature these rhythms are present in almost all organisms ranging from single-celled eukaryota, to plant and fungal life, on to higher order mammalian species such as man [1]. In real-world situations the underpinning circadian clock synchronises (entrains) to rhythmic cycles in the environment (zeitgebers) to ensure appropriate phasing and maximally beneficial temporal organisation of an organism’s behaviour and physiology. While several zeitgebers of the system such as feeding time, temperature, exercise, and social interaction have been found to produce phase changes, the most potent stimulus which circadian rhythms respond to is the recurring light/dark cycle [2]. In mammals, the circadian timing system is a complex, distributed system with a master pacemaker located at the suprachiasmatic nuclei of the hypothalamus (SCN) and other pacemakers present throughout the brain and the periphery [3]. Therefore, it is not surprising that circadian clocks are increasingly linked to health and wellbeing, with dysfunction being linked to common diseases (e.g. diabetes [4]), psychiatric disorders such as schizophrenia [5] and attention deficit hyperactivity disorder [6], and even to longevity [7].

One area of considerable public health importance is the extent to which human health is impacted by shift work. It is estimated that one in five workers work shift involving night work as part of their recurring work schedule [8]. Given that these workers will be exposed to light during the night shift, which would normally be expected to alter the circadian phase, it is likely that there will be considerable circadian system involvement in mediating health effects of shift work. There is accumulating evidence that shift work is associated with adverse health outcomes. In 2007 the International Agency for Research on Cancer (IARC) designated shift- work as a class 2A carcinogen concluding that ‘shift-work that involved circadian disruption is probably carcinogenic to humans’ based on ‘sufficient’ animal evidence but ‘limited evidence in humans’ [9]. A number of studies have also examined increased risk of cardiovascular disease (CVD) and shift-work [10]. There is evidence also which suggests an adverse association between shift-work and risk of metabolic syndrome [11, 12]. A recent study examining risk of multiple sclerosis (MS) in those that began shift-work at a young age revealed that there may be an association between the neuroinflammatory disorder and shift-work [13].

Aside from the longer-term health consequences, there are acute neurobehavioural outcomes associated with shift-work. Diminished executive functioning and maladaptive affective changes are among the psychological detriments associated with typical shift work regimes. Studies examining safety sensitive professions specifically have revealed that those employed as physicians, policemen, nurses, and pilots that are exposed to chronic “shift lag” perform significantly worse on measures of cognition and work related fatigue [14–16]. In addition to the cognitive effects experienced by shift-workers there is evidence to suggest that shift-lag may increase the risk of morbid affective change. Depression is one of the most common disorders found in working populations [17], with shift work being associated with increased risk of anxiety or depressed mood [18, 19]. Finally, and perhaps unsurprisingly, sleep complaints are a common concern among shift workers. In many individuals engaged in non-conventional patterns of work, mismatch between work roster and normally active hours on non-working days can interfere with productivity, social activity, and domestic responsibility. It is estimated up to 30% of shift-workers develop a similar collection of disturbances [20] which is severe enough to enter the clinical range in about 10% of shift-workers [21], in which case a distinct circadian rhythm sleep disorder known as shift-work disorder (SWD) is diagnosed.

Central to understanding these health and neurobehavioural consequences of shift work is understanding how the circadian system adapts to shift cycles. To date the data from most studies of shift workers indicate minimal entrainment of circadian rhythms to shift cycles [22]. Given that research in human subjects with regards to shift work is somewhat difficult and presents challenges in understanding the myriad factors that may influence shift work-related health outcome (eg. diurnal preference, work nature, number of year working shift, age at first shift work, nature of shift cycle [23], an argument may be made that the use of animal models may provide useful insight into the human situation and augment findings from human experimental and field studies. A number of studies to date have emphasised the “work” component of modelling shift work in rodents and report metabolic impairments that arise from rodent “shift work” [24, 25] but these effects may be dependent on the type of “shift work” intervention used [26]. In the current study we have focused on the light component of shift cycles in mice, as light is the dominant zeitgeber, and have examined entrainment of circadian rhythms to light/dark cycles that may mimic (in part, at least) that experienced by shift workers on fast forward rotating, fast backwards rotating and night shift workers. We have also examined some neurobehavioural outcomes in such animals in order to ascertain any potential links between circadian responses and resulting changes in behaviour and cognition.

## Methods

### Animals

Forty male CD-1 mice (8 – 12 wk old at the beginning of the experiment) were obtained from Harlan Laboratories (Leicestershire, UK). Animals were individually housed in polypropylene cages (29 × 13 × 12 cm) equipped with steel running wheels (11.5cm diameter). Food and water were available ad libitum and animals were maintained in a constant environment; ambient temperature of 21 ± 2°C, circulating air, constant humidity of 50 ± 10%. Mice were maintained in a 12-h light, 12-h dark cycle (lights on 0700; lights off 1900) with the exception of when experimental conditions demanded otherwise. The time at which the light switched on was defined as Zeitgeber time zero (ZT0). Manipulation of light-dark environment was achieved via an environmental isolation cabinet which allowed for complete control over exposure to photic stimuli. The interior of the cabinet was of a black and non-reflective material and the light luminance inside the cabinet was provided by standard fluorescent lighting and of low intensity (~50 lux at cage level) to avoid the development of cataracts in the albino mouse. All protocols were approved by the Research Ethics Committee at the National University of Ireland Maynooth (BSRESC-2011-0018) and licensed by the Department of Health and Children Ireland. All animals were treated in accordance with the Cruelty to Animals Act, 1876 and the SI No.17 – European Communities (Amendment of Cruelty to Animals Act, 1876) regulations, 1994 (European Directive 86/609/EC). All efforts were made to minimise the number of animals used in this study and any suffering or discomfort.

### “Shift work” protocols

Twenty-four mice were assigned to three different experimental cohorts in this study (n = 8 per group). The remaining sixteen animals were assigned to two control cohorts (n = 8 per group). The experimental conditions in this study were designed to mimic different patterns of rapidly rotating shift-work. Hence, three groups were selected to be put on shifting light-dark (LD) schedules which would resemble: (a) ‘forward-rotating’ or clockwise rotating shift-work patterns (Fwd); (b) ‘backwards -rotating’ or counterclockwise rotating shift-work patterns (Bck); and (c) within week alternating day and night shift-work patterns (Alt). In the Fwd protocol animals were exposed to 8 h phase delays every 2 d for six days followed by 2 d in constant darkness (DD) to mimic ‘days off’. In the Bck protocol animals were exposed to 8 h phase advances every 2 d for six days followed by 2 d in DD. In the Alt protocol animals were phase shifted by 12 h after 3 d on the equivalent of a day schedule. There were no “free” DD days in the Alt group. Timings of lights on/off for these schedules can be found in Table 1.

**Table 1:** **Schedules of shift work-like light/dark used in this study**

	+Lights on -Lights off		
	Fwd	Bck	Alt
Day 1	+1700-0500	+0700-1900	+0700-1900
Day 2	+1700-0500	+0700-1900	+0700-1900
Day 3	+2300-1100	+2300-1100	+0700
Day 4	+2300-1100	+2300-1100	−0700 + 1900
Day 5	+0700-1900	+1700-0500	−0700 + 1900
Day 6	+0700-1900	+1700-0500	−0700
Day 7	DD	DD	+0700-1900
Day 8	DD	DD	

All experimental animals were maintained on a 12:12 h LD cycle initially before commencing the shift-work component of the study for two weeks. After completing the respective shift-work protocols for five cycles of the shift work schedule, animals were released into DD for 2 wk to assess changes in free running circadian parameters. To avoid any additional phase shifts, animals that were released into DD for 2 d after each block of work or at the end of the shift work protocol were done so in the animals’ lights off phase. Following DD, the animals were then place into a stable 12:12 LD cycle for a further two weeks to assess their post-shift work entrainment. These protocols differ from, but also have some relationship to, those used to induce chronic jet-lag, which typically involve an advance of the LD cycle that recurs on a regular basis (eg. a 6h advance every 7 cycles as in Castanon-Cervantes et al, 2010, [27] or a 6h advance every 2 cycles as in Logan et al 2011 [28]). There are also some differences in chronicity of the paradigms used, with some chronic jet-lag protocols being applied over a shorter timeframe (eg. 20 days [28] as opposed to circa 40 days in the present study).

Of the two control groups, one was maintained on standard 12:12 h LD cycle (lights on 0700) for 5 wk before being released into DD for 2 wk. The second control group was maintained on a standard 12:12 h LD cycle like the previous control animals however after every 5 d on a normal LD cycle animals were exposed to 2 d in DD in order to control for any possible change due to DD exposure. As in the analysis of results no differences were found between these control groups, data were amalgamated and treated as one control condition. Control and shift work mice were weighed at baseline and each week in the shift-work condition, as well as in DD at the termination of the shift work intervention.

Locomotor activity (wheel running) was recorded via microswitches attached to the axis of the running wheels in the environmental cabinet. Activity was monitored continuously using Chronobiology Kit (Stanford Software Systems, CA, USA) which digitally recorded behavioural events for later analysis. Actograms from each animal were double-plotted over 48 h to clearly represent behavioural patterns which were created by collecting the sum of activity over 5 min intervals. Circadian period and waveform amplitude (rhythm power) were analysed using chi-squared periodogram function in the Chronobiology kit.

### Open field and novel object location testing

2 weeks following the completion of running wheel activity recording, animals from the control, Bck and Alt groups were tested in the open field test and also in the novel object location test (due to logistical reasons we were not able to test the Fwd group on these measures). For the open field test the arena was an aluminium circular area measuring 35 cm in diameter at the base of the arena (total arena area = 1017 cm^2^). The base of the arena was painted black to facilitate detection by recording equipment as the CD-1 mouse is an albino strain. The test room was illuminated at the same intensity as the colony room. Each mouse was placed in the centre of the open field, and its behaviour was observed for 5 min. The recording equipment was connected to a camera suspended above the arena with a wide enough focus to record all locomotor activity within the open field area. Ethovision (Noldus) was used to evaluate locomotor parameters such as distance travelled, velocity of each animal, and total amount of time spent mobile. To assess thigmotaxis the area was divided into two tracks defined by the outside area which was separated by a digitally imposed corridor which ran around the area 6 cm from the periphery, and the inner track which was the entire remaining area of the arena. The time spent by each animal in both corridors was recorded, in addition, locomotor factors (speed, distance etc.), the number of rearings (mouse standing on two hind paws not touching arena walls) and defecations were analysed by two independent observers from the Ethovision recordings. At the end of each test the whole area of the arena was sprayed clean with an ethanol solution and wiped with a dry paper towel removing all traces of mouse droppings and urine.

The novel object location task is a test used to assay differences in rodent exploratory behaviour and is based on the innate tendency of mice to explore moved objects for longer periods of time compared to non-moved ones. The protocol consisted of two experimental epochs each lasting 5 min. The first part of the test consisted of an exploratory trial in which animals were first exposed to the arena with two unfamiliar objects during which differences in exploratory behaviour functioned as an operational assessment of anxiety. The second trial took place 4 h after the initial trial and involved a second trial during which recognition of the object which underwent a novel spatial change measured animal cognition. The object location task took place in the same arena as the open field test. The task took place after animals had undergone the open field test. The objects chosen were a stone and a beaker lid with the latter being moved to a different point during the second trial. All objects were sufficiently heavy so that the mice did not move them during exploration. The amount of times each animal spent exploring both objects was recorded by two independent observers. Exploration was defined as the number of nose contacts or front paw touches each animal made with each object. Between trials both objects and the arena were wiped clean with an ethanol solution. Preference index was calculated for the recognition trial as PI = (N-F)/(N + F), where N is exploration of the novel location object, F is exploration of the familiar location object.

### PER1 and PER2 immunohistochemistry

Animals from the Bck, Alt and control groups were anaesthetised using halothane and euthanised via cervical dislocation at ZT6 and ZT 12 2 weeks following the open field and novel object location tests. Whole brains were harvested and immersion-fixed in 4% paraformaldehyde in 0.1 Μ phosphate buffered saline for 48 – 72 h, before being cryoprotected in 30% sucrose. Brains were frozen on dry ice and cut serially into coronal sections (30 μm) through the rostrocaudal axis of the SCN (Bregma -0.22 to -0.82) using a freezing-stage microtome (Leica). The location of the SCN was defined using the cortical features described in the Paxinos and Franklin mouse brain stereotaxic atlas [29]. Free floating SCN sections were used according to a standard ABC/Nickel DAB protocol [30]. Primary antibodies were for PER1 (1:500, Santa Cruz Biotechnology) and PER2 (1:1000, Santa Cruz Biotechnology). Stained sections were mounted onto gelatine coated slides, dried overnight, dehydrated in ethanol, and delipified in histoclear. Slides were cover slipped using Eukitt mounting medium (Sigma-Aldrich).

For analysis, microscopic images were captured using an Olympus BX51 microscope with an Olympus DP12 digital microscope camera interfaced with DP12-BSW imaging software. For each region of interest images were viewed at 100x magnification for identification and photography of regions. Immunostaining was quantified using ImageJ 1.43u (N.I.H., U.S.A.). Integrated density of immunostain was taken as a measure of immunoreactivity.

### Statistical analysis

Effects of shift work interventions on circadian and other parameters were assessed using mixed between within groups factorial ANOVAs or one way ANOVAs, as appropriate. All values are means ± standard error of the mean. P < 0.05 was deemed statistically significant. Where appropriate, Tukey HSD (for between groups analysis) or Bonferroni (for within subjects analysis) *post-hoc* testing was used to examine pairwise differences. Statistical Analysis was done with SPSS software (version 20, IBM corporation).

## Results

**Figure 1:**
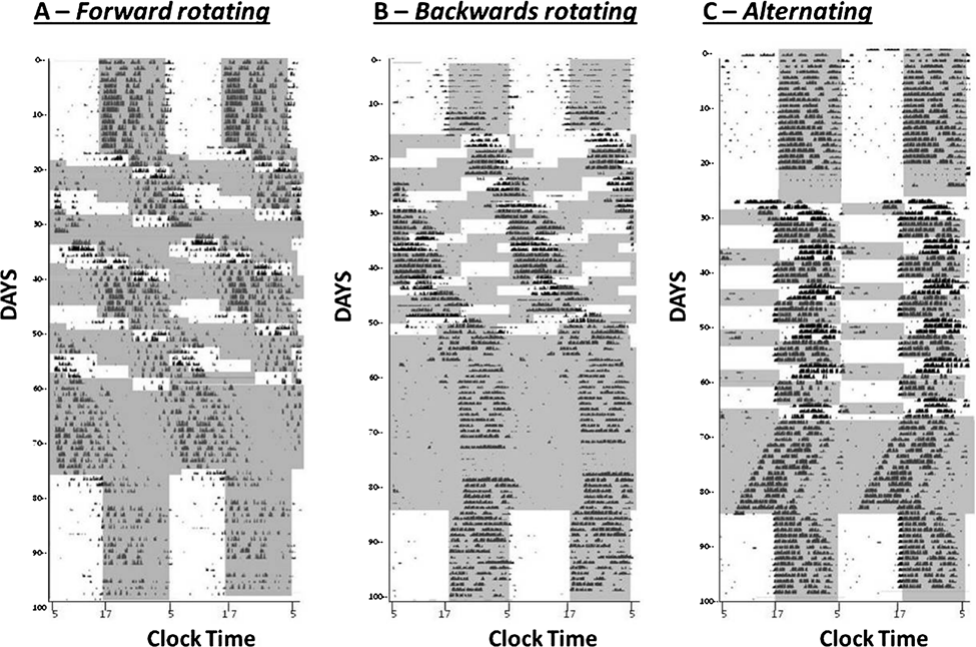
**Sample double plotted actograms from animals in the (A) Fwd, (B) Bck and (C) Alt groups.** The shading represents periods of darkness. The schedule entailed a stable light/dark cycle (12:12), five iterations of the “shift work” schedule, exposure to DD and then another stable light/dark cycle. Note the free-running-like patterns of activity adopted by the Fwd and Bck animals during the shift work protocols.

**Figure 2:**
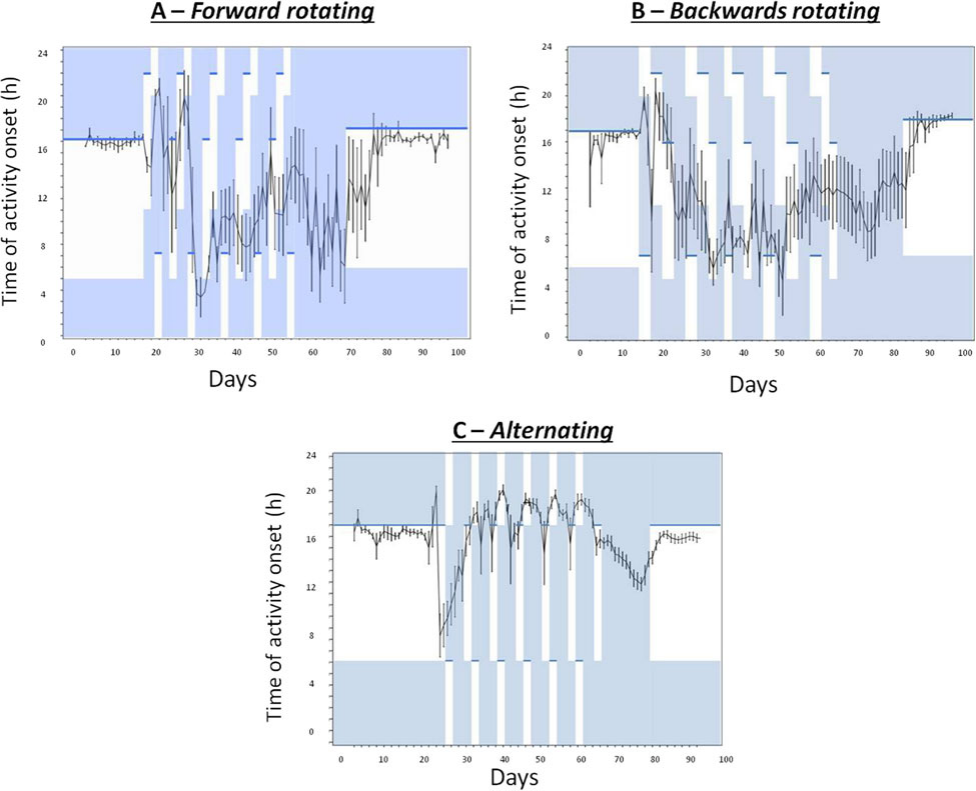
**Activity onsets across the three shift work-like schedules.** Plots representing the mean onsets of activity for the (**A**) Fwd, (**B**) Bck and (**C**) Alt groups throughout the stages of the running-wheel activity monitoring. The shaded area represents periods in darkness with the thickened horizontal line represents the onset of darkness.

**Figure 3:**
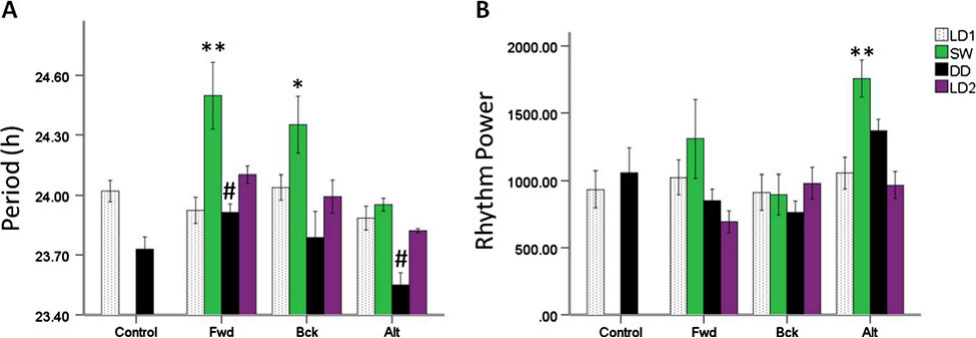
**Circadian period and rhythm power across the three shift work-like schedules.** Mean (**A**) period and (**B**) rhythm power during the phases of the behavioural intervention in the control, Fwd, Bck and Alt groups. LD1 refers to the initial period in a 12:12 LD cycle, SW for the last 3 weeks in under the appropriate shift work protocol, DD for constant darkness following the shift work protocol and LD2 for the 12:12 LD cycle at the conclusion of circadian rhythm monitoring. ** represents P < 0.01, * P < 0.05 compared to the initial LD values for that shift work group (Bonferroni post hoc test, # P < 0.05 compared to corresponding value for the control group (Tukey HSD test). N = 8 for each group.

Observation of actograms of animals from the Fwd and Bck intervention groups suggests that animals arrange locomotor rhythms in a similar fashion. Following normal as expected entrainment to the baseline LD cycle, upon exposure to the fast rotating schedules there appears to be initial attempts of the animals to track their onsets of activity to the onset of darkness (Figures 1 and 2). However, for the final three cycles of the shift work schedules there appears to be a minimal entrainment of the onsets of activity to the time of lights off, with the rhythms appearing to resemble that of a free running animal with a period of greater than 24 hours. The Alt group of animals appears to attempt to track the onset of darkness, with activity onsets showing a greater propensity to lock into the light/dark cycle than the Fwd or Bck groups (Figures 1 and 2). Analysis of rhythm period and power across the 3 shift work groups for the protocol stages of (1) the initial stable LD period (LD1), (2) the final three weeks of the shift work protocol (SW), (3) the period of DD following termination of the shift work schedule and (4) the period of stable 12:12 LD (LD2) at the end of circadian monitoring reveals a number of significant findings. For period, there is a main effect of protocol stage (F (3, 63) = 16.8, P < 0.001) and a main effect of shift work group (F (2,21) = 15.6, P < 0.001), but no group x stage interaction. Post-hoc analysis reveals that both the Fwd and the Bck groups showed lengthening of the period in the shift work phase compared to the LD or DD phases (Figure 3A). For rhythm power, there is a main effect of protocol stage (F (3,63) = 8.1) and a stage x shift work group interaction (F (6, 63) = 3.7, P < 0.01). Post-hoc analysis revealed that the Alt group showed a significant increase in the rhythm power in the shift work phase compared to in LD (Figure 3B). When comparing free-running periods in DD for the three shift work groups compared to that of controls there was a significant lengthening of period in the animals that had previously exposed to the Fwd protocol and a shortening of period in animals exposed to the Alt protocol compared to controls (Figure 3A). The animals previously exposed to the Alt protocol also showed an increase in their rhythm power in DD compared to controls (Figure 3B).

**Figure 4:**
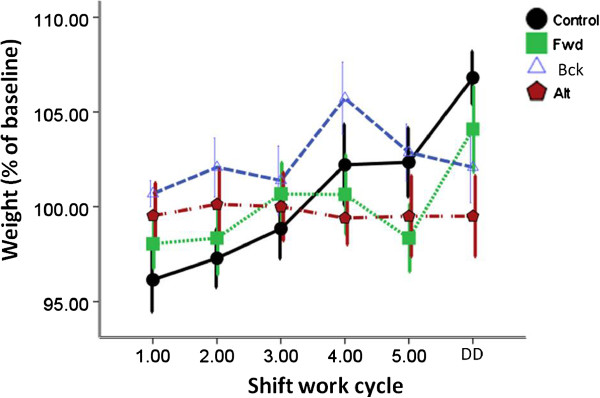
**Weight gain (expressed as a percentage of starting weight) throughout the “shift work” cycles.** N = 8 per group.

Examination of body weights revealed that there was a significant main effect of time on body weight (F (5, 180) = 7.3, P < 0.001) but also a time x group interaction (F (15, 180) = 3.5, P < 0.001; Figure 4). Further analysis revealed that the Alt group animals did not significantly gain weight during the course of the experiment, and at the final timepoint their body weight was significantly different to the control animals (P < 0.01). The Fwd and Bck animals did not differ significantly from controls or each other.

**Figure 5:**
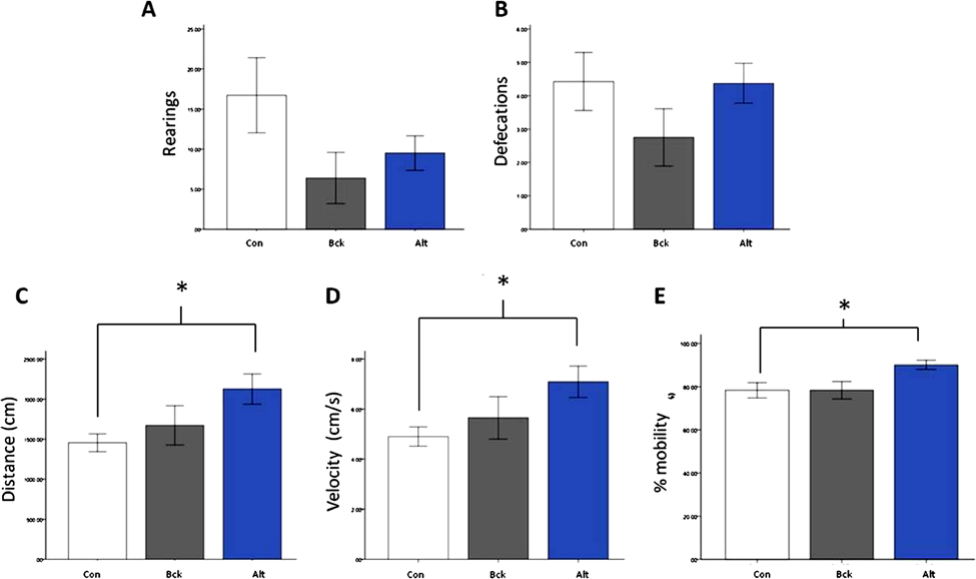
**Measures of activity in the open field test.** (**A**) number of rearing in the sessions; (**B**) number of defecations in the sessions; (**C**) overall distance travelled in the sessions; (**D**) velocity of movement in the sessions; (**E**) % of time spent mobile in the sessions. * = P < 0.05 compared to control, N = 8 per group.

**Figure 6:**
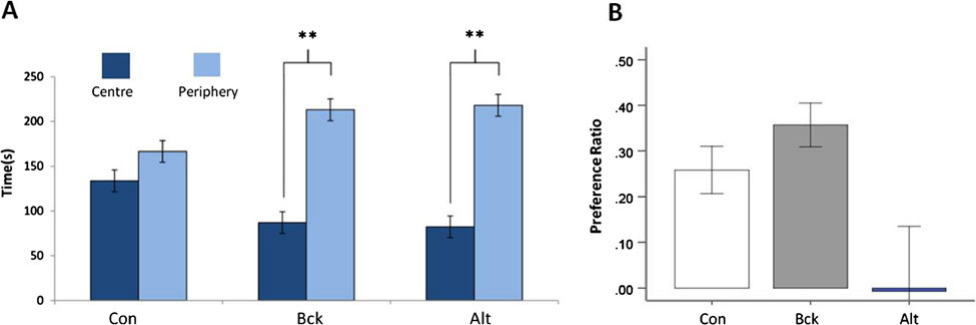
**Thigmotactic behavior and performance in the novel object location test.** (**A**) Measure of thigomotactic behaviour in the open field test, expressed as mean time spent in the central and peripheral zones of the arena; (**B**) performance on the novel object location task. The control and Bck animals showed significant preference for exploring the moved object, the Alt animals did not. N = 8 per group. ** = P < 0.01.

On the open field test, there were no groupwise differences on rearing or defecations. The Alt animals were found to travel further, have a higher velocity and spend more time mobile than the control animals (Figure 5). When open field thigomotaxis was examined there was a significant interaction effect between group and area (F (2, 40) = 10.42, p < 0.001). Alt and Bck animals displayed higher levels of thigomotaxis as evidenced by their marked preference for the periphery when compared to controls (Figure 6A). On the novel object location test the control and Bck animals display significant preference for the moved object, whilst the Alt animals did not (Figure 6B). There was not a significant difference on the total number of explorations of the objects (data not shown).

**Figure 7:**
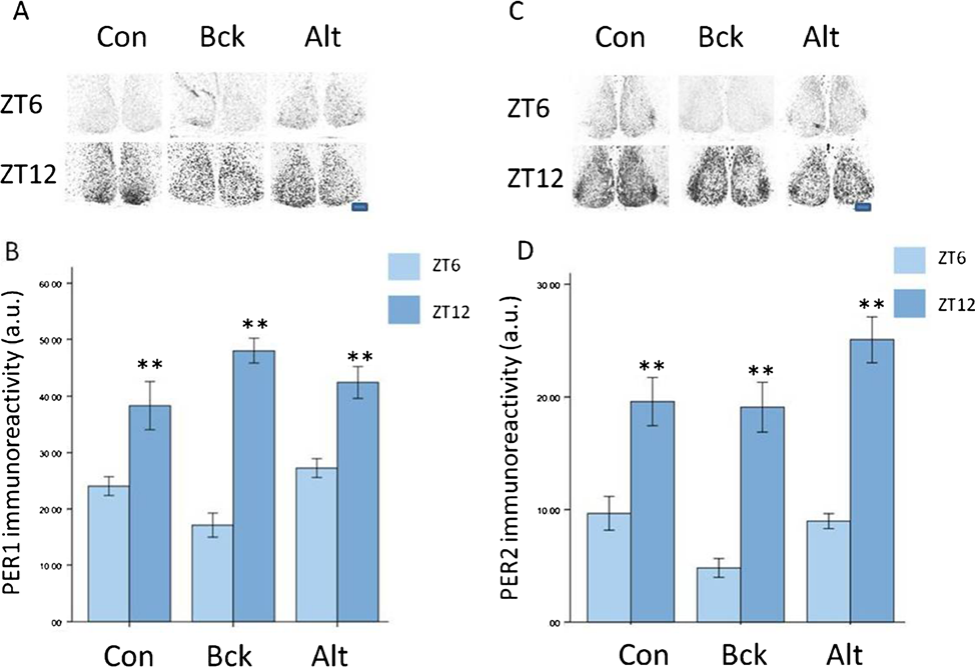
**PER1 and PER2 expression in the SCN.** (**A**) + (**B**) expression of PER1 in the SCN at ZT6 (predicted near nadir of expression) and ZT12 (predicted near peak of expression) in the SCN in the control, Bck and Alt groups. ** = P < 0.01 for ZT12 values compared to ZT6 values for each group. (**C**) + (**D**) expression of PER2 in the SCN at ZT6 (predicted near nadir of expression) and ZT12 (predicted near peak of expression) in the SCN in the control, Bck and Alt groups. ** = P < 0.01 for ZT12 values compared to ZT6 values for each group. Scale bar = 100 μm. N = 4 per timepoint per group.

When examining the expression of PER1 in the SCN, there was significantly more immunostaining for PER1 in the SCN at ZT12 than at ZT6 in each of the groups and no differences between the groups for ZT12 or ZT6 PER1 expression (Figure 7A,B). Likewise similar results were observed for PER2 immunostaining (Figure 7C,D).

## Discussion and conclusion

In the present study we exposed mice to different schedules of rapidly rotating shift work-like cycles of light and dark to assess circadian entrainment and neurobehavioural affects. We report differential entrainment to fast rotating shift cycles as opposed to a slower alternating night shift type paradigm. We also report that there may be longer term plastic changes in the circadian system that persist after the termination of shift work schedules, and that there also may be longer-term changes in neurobehavioural and cognitive parameters. The finding that on fast rotating schedules there appears to be minimal entrainment may be of interest as it has been hypothesised that in shift workers a similar lack of entrainment may serve as a mechanism in the long term preventing the maladaptive consequences of rhythms having to constantly re-entrain to rotating patterns of shift work [31]. Indeed re-entrainment is associated with internal circadian desynchrony at a molecular and physiological level [32, 33] and thus lack of entrainment is hypothesised to be beneficial with respect to reducing circadian insult and thereby facilitating health in the organism. There are preliminary findings indicating that in animals exposed to similar patterns of LD cycle rotation those that free-run rather than re-entrain have better outcomes after inoculation with a lung tumour inducing agent [28]. Of the three patterns of rotating shift-work examined in this study the Alt group animals were the only cohort to exhibit partial entrainment. This seems to suggest that with respect to entrainment tracking of the LD cycle is more responsive to 12 h phase shifts every 3-4 d than to 8 h phase shifts every 2 d which does not appear to facilitate entrainment at all. There have been suggestions that fast rotating schedules are better tolerated in shift workers than slower rotating schedules [34], and this may be due to the benefits of a lack of entrainment to faster schedules rather than partial entrainment to slower cycles. It has been postulated for human shift workers that adoption of an intermediate circadian phase with relation to the shift schedule may be beneficial [31, 35], and the current mouse results may be viewed in the Fwd and Bck groups as indicating that the animals are indeed adopting a type of compromise response to the fast rotating schedules to which full entrainment is not possible.

Analysis of behavioural circadian rhythm period length and power following the shift work schedules revealed that forward-rotating patterns of shift-work significantly lengthened period while the Alt group showed significantly shortened period length. These findings appear to suggest plastic changes in the circadian system in response to preceding cycles and are reminiscent of ‘period after effect’, a phenomenon seen in animals exposed to non-24 h light-dark cycles in which the long-term free-running period of rodent locomotor activity undergoes plastic changes [36]. Aton and colleagues [37] demonstrate also that after effects involve long-lasting period changes in the SCN in PER1 expression indicating SCN involvement in this mechanism. Our analysis of PER1 and PER2 in the SCN suggests no gross abnormalities in the expression of these factors, although a full characterisation across the circadian cycle would be required to detect any more subtle changes. Future experiments may examine how enduring effects on circadian period are in shift work paradigms. There is evidence that ex-shift workers still suffer from detriments to sleep [38] and long-term changes in circadian parameters may have a role to play in these effects.

Epidemiological evidence suggests that shift workers are at increased risk of becoming obese and developing illnesses such as diabetes and metabolic syndrome [11]. In line with prior human investigations, animal models of non-rotating night-work undertaken by Salgado-Delgado and colleagues have reported increased weight gain in shift-worker animals compared to control groups [23, 24]. Such findings are not uncontested however as a similar simulation of non-rotating shift-work in the rat rather reports attenuation of normal weight gain compared to controls [25]. These results may be explained by the differences in the amount of activity undertaken in the light on and off phases. Our present findings do not suggest that there is increased weight gain, indeed the Alt group animals appear to show attenuated weight as compared to controls (perhaps due to hyperactivity, as suggested by the open field test results). Given that previous studies have focussed on enforced “work” in non-rotating schedules, the present paradigm involves rotating cycles and voluntary activity, perhaps explaining the disparity. Further, we did not measure food intake. In a recent mouse model of sleep fragmentation it was found that after 14 days of enforced locomotor activity during animals sleep phase resulted in hyperphagia and impaired glucose tolerance but did not yield any increase in weight [39]. Therefore future work of rotating cycles may focus on food intake as well as body weight.

It is known that experimental models of circadian disruption or sleep fragmentation can adversely affect mood and exacerbate anxiety in man [40]. This is also reflected in the epidemiology which finds that shift-work is a significant risk factor for mood disorders in occupational groups [19]. Consequently we were interested in investigating if shift work-like rotating LD cycle manipulations produced changes in measures of anxiety-like behaviour. In rodent models assessing anxiety-like traits open field thigmotaxis (i.e. where exploration is predominantly restricted to the periphery) is considered an important indicator of anxious state. In the current study mice that had been exposed to rapidly rotating patterns of shift-work displayed significantly greater thigmotactic proclivity in the open field which was not observed in controls. This finding suggests that exposure to rotating patterns of shift-work causes an increase in anxiety-like behaviour. Importantly these differences were measured after circadian stress had been eliminated suggesting that these changes in affect possess some degree of chronicity. Future studies should examine different paradigms of anxiety-like behaviour as well as other affective domains such as anhedonia and behavioural despair in “shift work” animals.

Comparison of locomotor parameters in control and experimental animals exposed to the open field indicate that Alt mice travelled a significantly greater distance, moved significantly faster, and were hyperlocomotive compared to controls. We do not consider these findings representative of increased exploratory behaviour given (i) animals’ thigmotaxic tendency, (ii) rearing behaviour matched control levels, (iii) there was not increased object exploration in the novel object location task and (iv) during testing mice appeared noticeably more agitated compared to other groups. The apparent enhancement of locomotor activity in these animals may represent hyperactivity in a novel environment within the Alt group. Future work should address the mechanisms by which this change may occur. It is also interesting to note that in the novel object location test it was only the Alt group that failed to show a preference for exploration of the moved object. Again, there was no evidence for increased overall exploration in this group. It may be that the mechanisms that underpin the apparent hyperactivity also play a role in producing some cognitive deficits, although further studies investigating a comprehensive battery of cognitive tests would be required before any firm conclusions may be drawn. Numerous previous studies involving manipulation of circadian rhythms reveal learning and memory deficits on different batteries of cognition in rodents which are subjected to repeated phase shifts [41, 42] although our present results would appear to indicate that these effects may be long-lasting.

There are a number of important caveats and limitations to the current study to note. First is that we have used nocturnal mice and human shift workers are diurnal. Future work may use diurnal rodent models (eg. the Nile rat). However, one may argue that in terms of a moving zeitgeber, such as the shifting onset of light/dark, it may be the changing of the timing of the zeitgeber, and not the absolute relationship to the active/inactive phase that is important. Another important caveat is that we have not attempted to model the “work” component of shift work, in that wheel running was voluntary. Future studies may address the interaction between wheel running and “work” by locking exercise to the lights on or lights off components of any shift cycle. Further, given the key role melatonin may play (for example in the “light-at-night” hypothesis [43]) it is worth noting that CD-1 mice, like nearly all laboratory mouse species, do not synthesise melatonin, and so there may be differences in melatonin-producing rodents. Differences in rates of entrainment between mice and humans may also be important in using mouse models. Further there is the difficulty of recapitulating the complex series of social and behavioural factors that also shape human adaptation to shift work (eg. behaviour during free days, light exposure during the commute to and from work). Another important aspect to consider is the extent to which human shift workers are exposed to both “imposed” work-place related light, which in turn is superimposed against a background of the solar cycle and environmental natural light exposure. Thus, shift workers coming off the night shift do not transfer directly into darkness, rather they are exposed to morning sunlight on the commute home and in other social situations. This is in contrast to the marked transition between light and dark used in the present paradigms, and it may be argued that the present protocols for this reason may be more reflective of chronic jet-lag, rather than shift work *per se*. Development of a more naturalistic modelling of shift work in rodents and other models is a key challenge for this area. The availability of good data on the actual light exposure of shift workers will aid in the development of these models. However, taking all of these important caveats into consideration, the controlled experimental conditions afforded by animal experiments do suggest that further development of animal studies of shift work may have significant contributions to make to understanding the key question of how shift working affects health and performance.
